# Pros and Cons of Immediate Sequential Bilateral Cataract Surgery from a Patient Perspective: A Survey

**DOI:** 10.3390/ijerph20021611

**Published:** 2023-01-16

**Authors:** Iwona Obuchowska, Zuzanna Micun, Maryla Młynarczyk, Diana Anna Dmuchowska, Joanna Konopińska

**Affiliations:** Department of Ophthalmology, Medical University of Białystok, Kilińskiego 1 STR, 15-089 Białystok, Poland

**Keywords:** immediate sequential bilateral cataract surgery, ISBCS, cataract surgery, phacoemulsification, cataract, survey

## Abstract

The aim of the study was to analyze patients’ experiences with immediate sequential bilateral cataract surgery (ISBCS). An anonymous survey was sent by post to patients who underwent ISBCS between 1 May 2020 and 30 April 2022. A total of 195 participants completed the survey. Specifically, 94.6% of the respondents were satisfied with the possibility of having both eyes treated during one surgical procedure, 89.7% would choose ISBCS again if offered a choice, and 89.2% would recommend this procedure to their family members. ISBCS provided a subjective benefit for 84.6% of the respondents. The most commonly reported ISBCS-related problems were the necessity to sleep in a supine position (32.8%), inability to read shortly after the surgery (27.7%), having both eyes protected with a dressing (24.6%), and application of eyedrops to both eyes at the same time (17.4%). All patients were able to identify some pros of ISBCS, with the most common being single stay in the operating room (82.6%), lower number of visits to the clinic (62.6%), quick normalization of eyesight (61%), time savings for family members (54.9%), quick adjustment of reading glasses (32.3%), and economical aspects (23.6%). The perception of difficulties associated with ISBCS and the benefits offered by this procedure differed depending on patients’ gender, age, marital status, education, place of residence, occupational activity, level of care dependence, and everyday activities. Understanding patients’ opinions about ISBCS provides insight into the advantages and disadvantages of this procedure from a broader perspective.

## 1. Introduction

Immediate sequential bilateral cataract surgery (ISBCS) is a procedure during which a cataract is removed from both eyes of a single patient during the same surgery, in a “one eye after another” mode. This procedure is also referred to as “one-day/same-day bilateral cataract surgery” or “simultaneous bilateral cataract surgery” [1,2]. Alternatively, cataracts in both eyes can be removed sequentially, days, weeks, or even months apart, which is known as delayed sequential bilateral cataract surgery (DSBCS). The first documented ISBCS was performed in 1952 [3]. However, the procedure was conducted relatively rarely after that, only in highly specific indications, e.g., to improve eyesight in a patient with very poor baseline visual acuity (graded from light perception to 0.1), due to economic reasons—to regain a “functional vision”, in persons requiring general anesthesia which posed a systemic risk, or for logistic purposes [2]. Such an attitude resulted from concerns about hypothetical, potentially harmful complications, such as endophthalmitis, toxic anterior segment syndrome, corneal endothelial decompensation, and bullous keratopathy development or poor refractive outcome [4,5,6]. 

Due to improvements in the ophthalmological surgical technique, in particular, a widespread of phacoemulsification, the number of complications has decreased substantially, and cataract surgeries have become generally safer. This revoked interest in ISBCS. The immediate bilateral procedure offers patients with multiple benefits, including very rapid visual rehabilitation, a lower number of visits to the clinic, which is particularly important in the era of the COVID-19 pandemic, limiting operative stress to a single event, the necessity of only one general anesthesia, avoidance of interoperative anisometropia and decreased stereopsis, and higher cost-effectiveness (less commuting, time savings for family members and caregivers, less absence from work) [1,6,7,8]. Currently, ISBCS is conducted and increasingly accepted in many countries around the world, among others in Australia, Austria, Canada, China, Finland, United Kingdom, Iran, Israel, Japan, Turkey, South Africa, Spain, Sweden, Poland and, to a limited extent, in the United States [2,7,9]. 

While ISBCS, without a doubt, provides multiple benefits, it needs to be remembered that it still poses some potential risks, including intra- and postoperative complications in both eyes and blindness. This raises concern, not only among ophthalmic surgeons who perform ISBCS, but also among patients qualified for this procedure [10]. In this study, we attempt to evaluate patients` perspective on costs and benefits of ISBCS. Having searching available literature, we found a few studies investigating the opinions of patients subjected to immediate bilateral cataract surgery [11,12,13,14]. Despite that, they seem to focus on general satisfaction of the surgery’s results without paying enough attention to the sole procedure itself or subjective difficulties regarding pre- and postoperative periods. To fill this gap, we developed an anonymous survey determining patient experiences with ISBCS. We hope that the results based on this survey will allow ophthalmologists to weigh the advantages and disadvantages of ISBCS from a broader perspective.

## 2. Material and Method

This study was conducted with approval from the Bioethics Committee of the Medical University of Bialystok (APK.002.367.2022) in accordance with the ethical standards as laid down in the 1964 Declaration of Helsinki and its later amendments or comparable ethical standards.

The study was carried out at the Department of Ophthalmology, Medical University of Bialystok, a tertiary referral center for adults from Podlaskie Voivodeship (a population of approximately 1.2 million).

The study was carried out with a survey designed by the authors and distributed by post mail. The survey was sent at the same time to all adult patients who underwent ISBCS between 1 May 2020 and 30 April 2022. The patients received a letter with an anonymous questionnaire and a written request to complete it and return it to the Ophthalmology Department in the envelope attached to the letter. The envelope was addressed and had a postage stamp stuck on it, and the respondent’s task was only to return the envelope with the completed questionnaire into the mailbox. The survey was completely anonymous to ensure its credibility and confidentiality, eliminate bias, and reduce patient stress, especially when giving negative answers. Initially, a pilot study was conducted with a small group of patients after ISBCS (n = 10) to optimize the survey in terms of clarity and validity of questions, as well as the print size and the time necessary to fill in the questionnaire. The outcome of the validation was assessed as good. The questionnaires from a pilot study were included in the overall pool.

The minimum time between the surgery and completing the survey was three months. The survey consisted of two parts. The first part contained questions about the demographic characteristics of the patients, such as gender, age, marital status, place of residence, type of household and education, as well as about their everyday activities, including occupational activities and the level of care dependence. All items included in this part were closed-ended single-answer questions. Questions included in the second part referred to respondents’ satisfaction with the immediate bilateral surgery and their opinions about the difficulties and benefits associated with the procedure. This part included both closed-ended single-answer questions and multiple-choice questions. The survey consisted of a total of 17 questions. Participation in the survey was voluntary and fully anonymous. Each respondent provided consent for the use of the survey’s data for the purposes of research publication. The survey form can be found in the Supplementary Materials (Tables S1–S12).

During the analyzed period, ISBCS was carried out in 280 patients. Qualification for ISBCS took place during the pre-operation visit, within 1–2 weeks before surgery. All patients with binocular cataract and visual acuity in the better eye ≤0.6 (according to the criteria of the National Health Service in Poland) were qualified for ISBCS. The second inclusion criterion was the lack of obvious contraindications to simultaneous binocular surgery, such as: (1) low number of endothelial cells per square millimeter; (2) subluxation of the lens; (3) shallow anterior chamber; (4) narrow pupil or posterior synechia; (5) corneal haze; (6) closed angle glaucoma. The last criterion was the patient’s informed consent to perform surgery on both eyes at the same time. This process included a conversation between the surgeon and the patient, during which the doctor explained what a cataract is and methods of its treatment, all pros and cons of the surgery, described possible intra- and postoperative complications, as well as management and medical recommendations after surgery. In each case, the qualifying physician assessed the patient’s individual risk of surgery and presented the most optimal method of treatment based on a thorough binocular ophthalmological examination. Subsequently, the patient gave informed consent to ISBCS based on the obtained information. Each patient had the opportunity to freely ask questions, clarify all ambiguities and problems during the qualifying visit. Relatives attended the qualifying visit at the patient’s request. The surgery was performed as a single-day procedure. The patients were hospitalized for approximately 6–7 h; this time included hospital admission, preparation for surgery, surgical procedure, 2- to 3-h stay in the recovery room, and discharge home. At the time of the discharge, each patient received a document with postoperative recommendations and a prescription for three types of eyedrops containing antibiotic, corticosteroid, and non-steroid anti-inflammatory drug, respectively, along with instructions on how to apply them. Routinely, all patients were recommended to apply all three types of eyedrops three times a day. In line with the recommendations of the national consultant for ophthalmology, a standard follow-up schedule included two control visits, 24 h and two weeks post-surgery (https://www.pto.com.pl/wytyczne (accessed on 15 April 2021). However, additional control visits were scheduled whenever necessary. Additional anti-oedema eyedrops were prescribed to the patients who presented with severe corneal oedema during the first control visit.

A monofocal lens has been implanted in all operated patients to both eyes. No cases of intraoperative complications, such as rupture of the lens capsule or lens subluxation, were documented. None of the patients experienced severe postoperative complications, such as endophthalmitis, toxic anterior segment syndrome, corneal endothelial decompensation, and bullous keratopathy development or poor refractive outcome. The group of 280 respondents included 62 (22.1%) persons with a refractory error of more than +/− 3.0 diopters. This subgroup included 44 persons with myopia and 18 with hyperopia.

## 3. Statistical Analysis

Statistical analysis was carried out with the R package, version 4.2.1, with the statistical significance threshold set at *p* = 0.05. Age, the only numeric variable, was presented as median ± interquartile range (IQR). The other variables were summarized with basic descriptive statistics applicable to a given measurement scale. Relationships between the characteristics of the participants and survey responses were analyzed with the chi-square test or Fisher exact test, whichever was adequate for the answer distribution. Between-group comparisons of respondents’ age were carried out with the Wilcoxon test (for two independent groups) or the Kruskal–Wallis test (for more than two independent groups). Whenever the result of the Kruskal–Wallis test was statistically significant, a post-hoc test with Bonferroni correction was carried out. Normal distribution of age at various levels of categoric variables was verified with the Shapiro–Wilk test.

## 4. Results

Completed surveys were returned by 195 out of 280 patients who underwent ISBCS, which corresponded to 69.6%. The responses from those patients, referred to further as the studied group, were subjected to statistical analysis. The sociodemographic characteristics of the studied group are shown in Table 1.

Median ± interquartile range. In response to the question, “Are you satisfied with your decision to undergo immediate bilateral cataract surgery on the same day?”, 188 (96.4%) participants chose the answer “Yes”; the answer “No” was chosen by five (2.6%) respondents, whereas two (1.0%) patients chose the response “I cannot tell”. The respondents were also asked about their subjectively assessed bilateral eyesight after ISBCS. Responses to this question are summarized in Table 2.

Patients’ opinions regarding the most important disadvantages and benefits of ISBCS are shown in Table 3 and Table 4, respectively.

In response to the question, “If you could choose ISBCS again what would your decision be?”, 175 (89.7%) respondents chose the option “The same”, whereas the answers “Different” and “I cannot tell” were indicated by seven (3.6%) and 13 (6.7%) patients, respectively. However, asked if they decided to undergo ISBCS again in the era of the COVID-19 pandemic, up to 179 (91.8%) patients would choose the immediate bilateral surgery, and only three (1.5%) would make a different decision; the percentage of patients who were unable to respond unequivocally to this question was still 6.7%.

When asked, “Would you recommend the immediate bilateral surgery to your acquaintances and/or family members?”, 174 (89.2%) participants responded “Yes”, whereas six (3.1%) and 15 (7.7%) chose “No” and “I cannot tell” answers, respectively.

The analysis of associations between the sociodemographic characteristics of the studied group and responses about the pros and cons of ISBSS identified many statistically significant relationships.

### 4.1. Gender

Both male and female respondents pointed to a single stay in the operating room and therefore less stress as a primary advantage of ISBCS. Men chose this response significantly more often than women (90.7% vs. 77.5%, *p* = 0.03). Moreover, men significantly more often than women considered a quick return to regular activities of daily living, such as driving a car, or using a computer and mobile phone/smartphone, as a benefit of the immediate bilateral surgery (32.0% vs 17.5%, *p* = 0.031). Meanwhile, men less often than women pointed to avoiding interoperative anisometropia as a benefit of ISBCS (9.3% vs. 23.3%, *p* = 0.022). The latter response was the least often mentioned benefit of immediate bilateral surgery among male respondents. When it comes to women, the least frequently identified benefit of ISBCS was a quick return to work. This answer was chosen by 9.2% of women and 13.3% of men; the between-group difference was not statistically significant.

### 4.2. Age

The age was presented in years as median ±IQR. Respondents who more often pointed to applying eyedrops to both eyes at the same time and poorer bilateral eyesight immediately after the surgery were significantly older than other participants (76.0 ± 8.75 vs. 73.0 ± 15.0, *p* = 0.043) for bilateral eyedrop application, and 77 ± 12.0 vs. 72 ± 14.5, (*p* = 0.002) for eyesight deterioration. Moreover, significant differences were found in the age of patients who more often mentioned some specific benefits of the immediate bilateral surgery. Patients who chose (1) single stay in the operating room (75.0 ± 12.0 vs. 67.0 ± 17.0, *p* = 0.001), (2) lower number of control visits (75.5 ± 12.75 vs. 69.0 ± 15.0, *p* < 0.001), (3) lower number of visits to the clinic in the era of COVID-19 pandemic (76.0 ± 12.5 vs. 72.0 ± 14.0, *p* = 0.007), and (4) time savings for family members (75.0 ± 12.5 vs. 72.0 ± 14.25, *p* = 0.001) as benefits of the immediate bilateral surgery turned out to be significantly older than other participants. Meanwhile, those who pointed to (1) quick normalization of eyesight in both eyes (72.0 ± 13.0 vs. 76.5 ± 12.0, *p* = 0.001), (2) quick adjustment of reading glasses (70.0 ± 17.5 vs. 75.0 ± 12.0, *p* = 0.001), (3) quick return to work (59.0 ± 10.0 vs. 75.0 ± 12.0, *p* < 0.001), (4) quick return to regular activities of daily living (69.0 ± 15.0 vs. 75.0 ± 12.75, *p* = 0.002), and (5) avoiding interoperative anisometropia (66.0 ± 16.0 vs. 75.0 ± 12.0, *p* < 0.001) were significantly younger compared with other participants. 

### 4.3. Marital Status

Non-married (100%) and widowed persons (90.4%) pointed to a single stay in the operating room as a benefit of the bilateral surgery significantly more often than married individuals (76.9%, *p* = 0.037). Married persons (33.3%) significantly less often than widowed (47.9%) and non-married respondents (100%) considered a lower number of visits to the clinic in the era of the COVID-19 pandemic as a benefit of ISBCS (*p* = 0.002). Widowers significantly less often than non-married and married persons chose a quick return to regular activities of daily living, including driving a car and using a computer/mobile phone (11.0% vs. 40.0% vs. 29.9%, *p* = 0.003) and quick return to work (4.1% vs. 20.0% vs. 14.5%, *p* = 0.034) as a beneficial effect of the immediate bilateral surgery. Non-married persons significantly more often than other respondents pointed to economic savings as a benefit of the immediate bilateral procedure (80.0% vs. 21.4% of married respondents and 23.3% of widowers, *p* = 0.018). Time savings for family members and acquaintances assisting the patient in commuting to the hospital were most often appreciated among non-married (100%) and widowed persons (61.6%), whereas married respondents pointed to this benefit of the immediate bilateral surgery significantly less frequently (48.7%, *p* = 0.024).

The type of patient’s household (living alone, with family or friends) did not significantly affect the survey responses.

### 4.4. Place of Residence

Patients living in big cities (more than 500,000) and mid-sized towns (50,000 to 150,000) significantly more often than other respondents claimed that the immediate bilateral surgery had no disadvantages (*p* = 0.003). This response was chosen by 50.0% of big-city residents and 53.8% of those living in mid-sized towns.

Place of residence significantly affected the subjectively perceived benefits of ISBCS. While a single stay in the operating room and a lower level of resultant stress were the most frequently emphasized benefit of the procedure in all groups, residents of large towns (150,000 to 500,000) chose this answer significantly less often than other respondents (72.5% vs. 80.5% to 100%, *p* = 0.038). Quick return to work was significantly more often highlighted as a benefit of ISBCS by residents of mid-sized towns and big cities (15.4% and 21.7%, respectively) than those living in small towns and villages (7.3% and 1.4%, respectively, *p* = 0.001). Respondents from big cities and large towns significantly more often than other participants pointed to a quick return to regular activities of daily living (driving a car, using a computer and mobile phone, reading) (50.0% vs 34.8%, *p* = 0.03). This aspect was brought as a benefit of the immediate bilateral surgery by merely 12.2% and 18.6% of small-town and village residents, respectively. Meanwhile, the residents of villages (32.9%), small (29.3%), and mid-sized towns (30.8%) appreciated economic savings related to the immediate bilateral procedure significantly more often than respondents from big cities (10%, *p* = 0.009). Moreover, patients from small towns and villages significantly more often than those living in big cities pointed to time savings for family members as a benefit of ISBCS (*p* = 0.001). This response was chosen by 65.7%, 65.9%, and 61.5% of respondents from villages, small, and mid-sized towns, as compared with only 34.8% of patients living in big cities.

### 4.5. Education

Applying eyedrops to both eyes at the same time was significantly more often brought as a primary ISBCS-related inconvenience by persons with primary education (35.1% vs. 12.2%, 18.2%, and 7.0% of those with vocational, secondary, and higher education, respectively, *p* = 0.007). Education also exerted a significant effect on the subjective perception of multiple ISBCS-related benefits. Patients with secondary and higher education more often considered quick adjustment of reading glasses (39.4% and 44.2%, respectively), quick return to work (22.7% and 14.0%, respectively), and quick return to regular activities of daily living (33.3% and 32.6%, respectively) as benefits of the immediate bilateral procedure. Meanwhile, quick adjustment of reading glasses was considered as a benefit by 29.7% and 14.3% of respondents with primary and vocational education, respectively (*p* = 0.009), quick return to regular activities of daily living by none and 18.4%, respectively (*p* < 0.001), and quick return to work by none participant from either group (*p* < 0.001). Economic savings were pointed as a benefit by 48.6% of respondents with primary education, 22.4% and 21.2% of those with vocational and secondary education, respectively, and only 7.0% of patients with higher education (*p* < 0.001). Patients with primary and vocational education (70.3% and 63.3%, respectively) appreciated time savings for family members significantly more often than those with secondary and higher education (48.5% and 41.9%, respectively, *p* = 0.03).

### 4.6. Independence in Self-Care

Postoperative eyesight deterioration in both eyes was significantly more often pointed out as a problem by fully care-dependent persons (52.2%) than by partly care-dependent and fully independent respondents (27.9% and 18.6%, respectively, *p* = 0.002). Care-dependent patients appreciated a lower number of control visits significantly more often than independent respondents (82.6% vs. 56.6%, *p* = 0.032). Time savings for family members and acquaintances assisting patients in commuting to the clinic were significantly more often brought out as a benefit of the immediate bilateral surgery by fully and partly care-dependent respondents (69.6% and 79.1%, respectively) than by those being fully independent (44.2%, *p* < 0.001). Meanwhile, independent persons appreciated quick return to regular activities of daily living (29.5%) and quick return to work (15.5%) significantly more often than dependent respondents (*p* = 0.012 and *p* = 0.004, respectively). Quick return to regular activities of daily living was chosen as a benefit of ISBCS by 11.6% and 8.7% of partly and completely care-dependent persons, respectively, whereas quick return to work by none patient from either group.

### 4.7. Driving a Car

Non-drivers (persons who did not drive a car on a daily basis) significantly more often than drivers considered a single stay in the operating room (87.1% vs. 72.7%, *p* = 0.038), lower number of control visits (68.9% vs. 43.6%, *p* = 0.002), time savings for family members (63.6% vs. 32.7%, *p* < 0.001), and economic savings (27.3% vs. 12.7%, *p* = 0.033) as benefits of the immediate bilateral surgery. Meanwhile, drivers significantly more often than non-drivers appreciated quick return to regular activities of daily living, including driving a car (54.5% vs. 9.8%, *p* < 0.001) and quick return to work (29.1% vs. 3.0%, *p* < 0.001).

### 4.8. Using a Computer/Mobile Phone

Using a computer or mobile phone on a daily basis did not exert a significant effect on patients’ opinions about ISBCS-related difficulties but significantly influenced their opinions about the benefits of the procedure.

Frequent users of a computer/laptop significantly more often than other patients considered quick adjustment of reading glasses (50.0% vs. 22.4%, *p* < 0.001), quick return to regular activities of daily living (44.3% vs. 11.2%, *p* < 0.001), quick return to work (25.7% vs. 2.4%, *p* < 0.001), and avoiding interoperative anisometropia (31.4% vs. 10.4%, *p* = 0.001) as benefits of the immediate bilateral procedure. Meanwhile, patients who did not use a computer on a daily basis significantly more often than others appreciated time savings for family members (60.8% vs. 44.3%, *p* = 0.038) and economic savings (29.6% vs. 12.9%, *p* = 0.014).

Persons frequently using their mobile phones/smartphones significantly more often than other patients pointed to quick normalization of bilateral eyesight (67.5% vs. 50.0%, *p* = 0.024), quick adjustment of reading glasses (39.0% vs. 20.8%, *p* = 0.014), quick return to regular activities of daily living (31.7% vs. 8.3%, *p* < 0.001), and quick return to work (15.4% vs. 2.8%, *p* = 0.012) as beneficial effects of ISBCS. In turn, patients who did not use their mobile phones on a daily basis significantly more often than others appreciated a lower number of control visits (73.6% vs. 56.1%, *p* = 0.022) and time savings for family members (70.8% vs. 45.5%, *p* = 0.001).

The respondents were also divided into two groups, those involved in daily activities requiring good eyesight, such as using a laptop, mobile phone, and/or reading a lot (n = 153), and persons involved in none of the activities mentioned above (n = 42). The “visually active” patients, who needed good near vision, significantly more often than other respondents complained about the bilateral deterioration of eyesight after the surgery (19.6% vs. 42.9%, *p* = 0.004). Compared with other respondents, those patients also significantly more frequently pointed to quick adjustment of reading glasses (37.9% vs. 11.9%, *p* = 0.003) and quick return to regular activities of daily living (27.5% vs. 7.1%, *p* = 0.010) as primary benefits of ISBCS. In turn, the less “visually active” persons significantly more often emphasized a lower number of control visits (78.6% vs. 58.2%, *p* = 0.025) and time savings for family members (71.4% vs. 50.3%, *p* = 0.024) as primary cons of the immediate bilateral procedure.

### 4.9. Occupational Status

Postoperative deterioration of eyesight in both eyes was reported as an immediate bilateral surgery-related problem by 28.2% of retirement/disability pension recipients without an additional source of income and 18.8% of occupationally active retirement/disability pension recipients, but only 4.3% of full-time employees (*p* = 0.029). Difficulty reading shortly after the surgery was, in turn, significantly more often mentioned as a problem by full-time employees than by other respondents (60.9% vs. 23.1% and 25.0% of retirement/disability pension recipients without an additional source of income and occupationally active retirement/disability pension recipients, respectively, *p* = 0.002). Retirement/disability pension recipients, both those without an extra source of income and occupationally-active ones, significantly more often than full-time employees appreciated a single stay in the operating room (87.2% and 87.5%, respectively vs. 47.8%, *p* < 0.001), lower number of control visits (68.6% and 56.2%, respectively vs. 26.1%, *p* 0.001), lower number of visits to the clinic in the era of COVID-19 pandemic (44.2% and 43.8%, respectively vs. 13.0%, *p* = 0.017), and time savings for family members (60.3% and 56.2%, respectively vs. 17.4%, *p* = 0.001) as benefits of ISBCS. In turn, full-time employees significantly more often than retirement/disability pension recipients without an additional source of income and occupationally active retirement/disability pension recipients considered quick adjustment of reading glasses (69.6% vs. 26.9% and 31.2%, respectively, *p* < 0.001) and avoiding interoperative anisometropia (52.2% vs. 12.8% and 18.8%, respectively, *p* < 0.001) as the procedure-related benefits. Quick return to work was most often considered as a benefit of ISBCS among full-time employees (79.6%), followed by occupationally active retirement/disability pension recipients (25.0%), and only a single (0.6%) retirement/disability pension recipient without an additional source of income (*p* < 0.001). Full-time employees and occupationally active retirement/disability pension recipients significantly more often than non-working respondents mentioned a quick return to regular activities of daily living as a benefit of the immediate bilateral procedure (52.2% and 50.0%, respectively vs. 16.0%, *p* < 0.001). Retirement/disability pension recipients without an additional source of income significantly more often than other participants pointed to economic savings as a beneficial effect of ISBCS (28.2%). This benefit was appreciated by only 8.7% of full-time employees and none of the occupationally active retirement/disability pension recipients (*p* = 0.005).

The exact data illustrating our results were collected in the twelve tables which can be found in Supplementary Materials(Tables S1–S12).

## 5. Discussion

The aim of the present study was to analyze the opinions and experiences of patients subjected to ISBCS because of bilateral cataract and to identify subjective pros and cons of the procedure. The majority of the patients were satisfied with their decision to undergo the immediate bilateral cataract surgery and declared that having a choice between the immediate and sequential procedure, they would again choose the former. While all respondents benefited from the immediate bilateral surgery, 84% also experienced some inconveniences. Pros and cons of the procedure brought by the patients depended on their gender, age, marital status, education, place of residence, care dependence, activity in everyday life, and occupational activity. We have identified only a few previous studies paying attention to the satisfaction of patients operated on for cataract with the ISBCS procedure. However, none of those studies analyzed patients’ opinions about ISBCS in correlations with the sociodemographic characteristics of the respondents [11,12,13,14].

A high proportion of our patients, up to 96.4%, were satisfied with their decision to undergo immediate bilateral cataract surgery. This percentage is similar to the one reported by Sarikkola et al. [11], according to whom up to 96% of operated patients had a positive or quite positive opinion about ISBCS, and 89% declared that they could safely return home after the procedure. Given a choice once again, up to 89.7% of our respondents would choose ISBCS, and only 3.6% would decide on another procedure; the remaining respondents could not decide their choice. In a study conducted in Northern California, up to 96% of patients after ISBCS would choose this procedure again, compared with 80% of patients who would opt for DSBCS again [12]. We also asked our respondents about the influence of the COVID-19 pandemic on their decision to undergo immediate bilateral surgery. Up to 91.8% of the patients stated that they would choose ISBCS again, even if the number of SARS-CoV-2 infections at the time of qualification and treatment was high, and only 1.5% would not decide on this procedure under such circumstances. The spread of the COVID-19 pandemic was a turnover point at which the rate of ISBCS procedures increased compared to DSBCS. To reduce the number of hospital visits and mitigate the risk of SARS-CoV-2 infection, many ophthalmology centers started offering patients the immediate bilateral procedure, as associated with a lower risk of the virus transmission, and the response was generally positive [15,16,17]. ISBCS was introduced to our clinic in 2019 after the procedure started being reimbursed by the National Health Service in Poland. Initially, the number of immediate bilateral surgeries was very low, but the COVID-19 pandemic contributed to a substantial increase in the number thereof. According to a growing number of experts, in the era of the SARS-CoV-2 pandemic, cataract surgeries for the fellow eye will be routinely performed on the same day as for the first eye [8]. A survey conducted among patients who were yet to be operated on for cataract showed that up to 45% of the respondents would request their procedure to be ISBCS [18]. This proportion should be considered very high, given that many patients make an informed decision about undergoing immediate bilateral cataract surgery no earlier than after a conversation with a physician during the qualification visit. According in the recent study [19], SARS-CoV-2 is probably associated with increased postoperative mortality and pulmonary complications, which justifies the urge to reduce the number of surgical procedures among the elderly.

Up to 89.2% of our respondents would recommend ISBCS to their family members and acquaintances. This percentage is within the 79% to 94% range reported in previous studies [11,12,17]. Only 3.1% of patients participating in our survey would not recommend the immediate bilateral procedure to their relatives, with another 7.7% being unable to provide an unequivocal response to this question. Meanwhile, a previous study showed that only 68% of patients after DSBCS would recommend this procedure to their friends and family members [12].

The part of our survey which referred to problems and benefits the respondents experienced within a short period after ISBCS provided interesting information. The problems were reported markedly less often than the benefits, and 15.4% of the respondents declared that they found no inconveniences related to ISBCS. The most common complaint, reported by one-third of the respondents, was difficulty sleeping in a supine position during the initial few days after the surgery. Indeed, we request that patients sleep on their backs to avoid inadvertent compression of operated eyes. Approximately 25–28% of the patients complained that after the surgery, their eyesight in both eyes temporarily deteriorated and/or they were unable to read. We assume that this problem was, among other causes, associated with more pronounced postoperative corneal oedema in patients with more severe cataract and slightly weaker endothelium. However, it must be stressed that patients with a low number of endothelial cells per square millimeter were not qualified for the immediate bilateral procedure. The deterioration of eyesight after the surgery was reported more often by older persons, which was probably associated with greater cataract severity in those patients than in younger individuals. Care-dependent respondents were another group of patients for whom bilateral eyesight deterioration after the surgery constituted a problem. Due to their physical disability or other limitations, good eyesight is vital for those persons. Fortunately, the problems with eyesight resolved quickly, and most patients regained functional vision in at least one eye within 1–2 weeks post-surgery.

Having both eyes protected with a dressing at the same time, reported as an inconvenience by 25% of the respondents, was not as serious problem as could be expected, as at least one eye in each patient was protected with a transparent cover, not to impede the vision. Moreover, the dressing was removed as early as the next morning after the procedure. Applying eyedrops to both eyes simultaneously constituted a problem for 17% of the respondents. Bilateral application of eyedrops may, without a doubt, pose a challenge, but the proportion of patients reporting this procedure as a problem was relatively low. Moreover, applying eyedrops to only one eye after DSCBS would unlikely be considered less challenging. Applying eyedrops to both eyes simultaneously was significantly more bothersome for older persons. The procedure requires accuracy and manual dexterity, which are usually lacking in the elderly. The application of eyedrops might constitute a particular problem for older persons living alone. However, even older patients staying with their families are usually left alone during the daytime when others are at work; meanwhile, in line with postoperative recommendations, eyedrops should be applied three times per day.

Only 3% of the respondents complained of irritation and tearing in both eyes. In the case of 4% of the participants, the inability to drive a car within a short period after the surgery was an issue. However, it should be remembered that the problem, probably even more severe, would also occur if the cataract was removed from only one eye at a time. Given that up to 96.4% of the patients subjected to ISBCS were satisfied with the procedure, the occurrence of postoperative problems and burdensomeness thereof do not seem to limit its applicability.

The benefits of ISBCS definitively outweighed the problems related to the procedure. All our respondents were able to identify at least 2–3 benefits of the immediate bilateral operation. The most often reported benefit (83%) was only a single stay in the operating room and the resultant lower level of stress. Other frequently mentioned pros of the immediate bilateral procedure included a lower number of visits to the clinic, particularly appreciated in the era of the COVID pandemic (63%), quick normalization of eyesight (61%), and time savings for family members and acquaintances who assisted the patient in commuting to hospital and postoperative care (55%). Other, less frequently listed albeit still important benefits included quick adjustment of reading glasses (32%), economic savings for patients and their families (23%), and quick return to regular activities of daily living, such as driving a car, using computer and mobile phone (23%).

For 18% of the respondents (n = 35), a benefit of the immediate bilateral procedure was avoiding interoperative anisometropia. The entire group of 280 patients subjected to ISBCS included 22.1% of persons (n = 62) with moderate to high refractory errors (>3 diopters). If qualified for DSBCS, those patients would face a problem of interoperative anisometropia. Notably, our survey was anonymous; thus, it is unclear what the actual proportion of patients with refractory errors among the respondents was. Assuming the proportion was the same as for the entire operated group, the survey would include 43 persons with moderate to high refractory errors. If this assumption is correct, 81% of those persons would consider avoiding interoperative anisometropia and decreased stereopsis as a benefit of ISBCS. Although this estimation is likely biased, the proportion of patients with myopia or hyperopia who brought this benefit of ISBCS and appreciated the possibility of obtaining spectacle independence, especially for distance vision, immediately after the immediate bilateral surgery, was probably high.

The perception of ISBCS-related benefits was strongly modulated by multiple sociodemographic factors. Older respondents, retirement/disability pension recipients, care-dependent patients, those living alone and/or in small towns/villages, as well as non-drivers, more often brought a lower number of visits to the clinic and economic savings for themselves and their family members as benefits of ISBCS. Thus, the benefits of the immediate bilateral procedure were primarily appreciated by persons who, for various reasons, required the assistance of others; for those patients, removing the cataract from both eyes during a single procedure was beneficial in both logistic and economic contexts. Meanwhile, worse educated and non-married persons pointed to economic savings as a primary benefit of the immediate bilateral surgery procedure.

On the other hand, younger respondents, those married, occupationally active, care independent, and active in everyday life, as well as residents of big cities, males, and better-educated individuals, more often appreciated quick normalization of vision, quick adjustment of spectacles for near work, driving car, reading, using computer and smartphone. Persons with secondary and higher education, those driving a car, and younger individuals more often pointed to a quick return to work as a primary benefit of the immediate bilateral procedure. This beneficial effect of ISBCS was also more often appreciated by male respondents, residents of big cities, and care-independent persons. The fact that men constituted two-thirds of occupationally active persons participating in our survey might explain why they appreciated quick return to work more often than female respondents. The same might refer to the place of residence, as nearly two-thirds of occupationally active respondents lived in mid-sized towns and big cities. Finally, care-dependent persons are unlikely to be occupationally active, which explains why contrary to care-independent respondents, they did not point to a quick return to work as a benefit of the immediate bilateral procedure.

Many benefits of ISBCS identified herein have already been reported previously, also in the studies analyzing the pros of the immediate bilateral procedure from the operator’s perspective [1,6,7,8,20,21,22]. However, our analysis based on the subjective opinions of the operated patients provides a novel insight into the problem in question. Our survey enabled us to identify the main difficulties experienced by patients subjected to ISBCS, along with the subjectively perceived benefits of the procedure. This knowledge might be used during conversations with persons eligible for surgical treatment of cataracts in both eyes, improving patient–physician communication, allowing patients to understand medical information better, and making ophthalmic surgeons more sensitive to patients’ problems. It should be emphasized that the information obtained from the surveys is of great practical value. They can help to familiarize new patients with the specific difficulties of the ISBCS procedure in detail and to guide them on how to deal with these disadvantages. Presentation of both positive and negative experiences of people operated on with this method can reassure the patient and help make a more conscious decision to operate on both eyes at once. It is worth mentioning that familiarizing patients with the real experiences of people who have already been operated on may be the most effective method of communication between the patient and the ophthalmic surgeon.

This study had some limitations that need to be considered. First, the respondents were operated on by a few different surgeons; theoretically, this might influence the outcome of the procedure and, thus, the patient’s perceptions [23]. However, it needs to be emphasized that all the operators were experienced and skilled surgeons, as only such are allowed to perform ISBCS at our clinic. In our opinion, the subjective outcome of the procedure perceived by the patients was influenced more by the type and severity of cataract, which varied from patient to patient and from eye to eye within the same patient. Unfortunately, due to the anonymous character of the survey, we could not analyze its results according to the clinical characteristics, such as preoperative visual acuity, type of cataract or ophthalmic comorbidities, e.g., glaucoma or age-related macular degeneration. However, the primary objective of this study was to analyze the subjectively perceived pros and cons of ISBCS, which were less likely influenced by clinical variables. While maintaining the original assumptions, we did not want to excessively expand the scope of research, so that the large amount of data does not obscure the essence of the problem we focused on. However, when analyzing the data from the surveys, we noticed that it would be interesting to correlate patients’ opinions with their local condition, in particular, with accompanying eye diseases. Thus, a relationship between the subjective opinions of patients after ISBCS and their clinical condition, whether local or systemic, prior to the procedure would be the direction for our future research. Such a study is planned to be conducted at our center.

The participation rate In our study was very high, up to 70%. However, 30% of the operated patients refused to participate in the survey. It is unclear whether the lack of participation was caused by logistic reasons, reluctance to complete the survey, laziness, or perhaps lower satisfaction with ISBCS. However, it should be stressed that the survey was fully anonymous, and persons being less satisfied with a medical procedure are usually more eager to participate in studies of this kind.

## 6. Conclusions

ISBCS is assessed positively by patients operated on with this method. Most patients would choose this procedure once again and recommend it to their family members and acquaintances. ISBCS should be preferred over DSBCS, especially in the era of the COVID-19 pandemic. Immediate bilateral surgery is not associated with many inconveniences and provides patients with multiple subjective benefits. All patients subjected to ISBCS could identify some beneficial effects of the procedure. The subjective benefits varied depending on the respondent’s gender, age, marital status, education, place of residence, occupational activity, care dependence, and activity level.

## Figures and Tables

**Table 1:** Sociodemographic characteristics of the studied group of patients subjected to immediate sequential bilateral cataract surgery.

Characteristic	Total Group n = 195	% of Group
Sex		
Female	120	61.5
Male	75	38.5
Age, Median ±IQR	74.00 ± 13.5	-
Marital status		
Unmarried	5	2.6
Married	117	60.0
Widowed	73	37.4
Living with		
Family	146	74.9
Friends/Partner	5	2.6
Alone	44	22.5
Place of living		
Village	70	35.9
City to 50 k citizens (small city)	41	21.0
City 50–150 k citizens (medium city)	13	6.7
City 150–500 k citizens (big city)	69	35.4
City over 500 k citizens (very big city)	2	1.0
Education		
Primary	37	19.0
Vocational	49	25.1
Secondary	66	33.8
Higher	43	22.1
I am (in life)		
Completely unaided	129	66.1
Partly unaided	43	22.1
Completely dependent	23	11.8
I drive by car		
Yes	55	28.4
No	132	68.0
Occasionally	7	3.6
Daily activity		
I use computer	70	35.9
I use mobile phone/smartphone	123	63.1
I read much	79	40.5
None of the above	42	21.5
Professional activity		
Non-working pensioner	156	80.0
Working pensioner	16	8.2
Working professionally	23	11.8
Unemployed	0	0.0

**Table 2:** Patients’ subjective opinions about their bilateral eyesight after immediate sequential bilateral cataract surgery.

How Do You Assess Your Vision at the Moment?	Number of Patients (%)
Very good in both eyes	123 (63.1)
Good in both eyes	44 (22.5)
Very good in one eye and good in the second eye	5 (2.6)
Very good/good only in one eye (bad in the second eye)	18 (9.2)
Bad in both eyes	5 (2.6)
I can’t say	0 (0.0)

**Table 3:** Patients’ opinions about disadvantages and difficulties related to immediate sequential bilateral cataract surgery (listed by frequency).

Disadvantages/Difficulties	Number of Patients (%)
Necessity to sleep on the back after surgery	64 (32.8)
Inability to read shortly after surgery	54 (27.7)
Both eyes covered with dressing	48 (24.6)
Poor vision in both eyes immediately after surgery	48 (24.6)
Necessity of application of drops to both eye at the same time	34 (17.4)
Inability to drive shortly after surgery	8 (4.1)
Lacrimation or irritation of both eye	6 (3.1)
There were no difficulties	30 (15.4)

**Table 4:** Patients’ opinions about the advantages and benefits of immediate sequential bilateral cataract surgery (listed by frequency).

Advantages/Benefits	Number of Patients (%)
One stay in the operating room—one stress	161 (82.6)
Limiting the number of control visits in hospital	122 (62.6)
Quick recovery good vision in both eye	119 (61.0)
Saving time of family/friends	107 (54.9)
Fewer visits in hospital during COVID-19 pandemic	79 (40.5)
Quick selection of glasses for reading	63 (32.3)
Cost effectiveness for patients and their family	46 (23.6)
Quick return to daily activities, computer use, driving	45 (23.1)
Avoiding anisometropia in patients with refractive errors	35 (17.9)
Prospect of quick return to professional activity (work)	21 (10.8)

## Data Availability

All materials and information are available upon an e-mail request on corresponding author.

## Supplementary Materials

The following supporting information can be downloaded at: https://www.mdpi.com/article/10.3390/ijerph20021611/s1, Table S1: Association between the gender of the respondents and their answers to the questions from the survey. Data in the table refer to positive answers to the individual questions; Table S2: Association between age of the respondents and their answers to the questions from the survey; Table S3: Association between themarital status of the respondents and their answers to the questions from the survey. Data in the table refer to positive answers to the individual questions; Table S4: Association between themarital status of the respondents and their answers to the questions from the survey. Data in the table refer to positive answers to the individual questions; Table S5: Association between themarital status of the respondents and their answers to the questions from the survey. Data in the table refer to positive answers to the individual questions; Table S6: Association between themarital status of the respondents and their answers to the questions from the survey. Data in the table refer to positive answers to the individual questions; Table S7: Association between themarital status of the respondents and their answers to the questions from the survey. Data in the table refer to positive answers to the individual questions; Table S8: Association between themarital status of the respondents and their answers to the questions from the survey. Data in the table refer to positive answers to the individual questions; Table S9: Association between themarital status of the respondents and their answers to the questions from the survey. Data in the table refer to positive answers to the individual questions; Table S10: Association between themarital status of the respondents and their answers to the questions from the survey. Data in the table refer to positive answers to the individual questions; Table S11: Association between themarital status of the respondents and their answers to the questions from the survey. Data in the table refer to positive answers to the individual questions; Table S12: Association between themarital status of the respondents and their answers to the questions from the survey. Data in the table refer to positive answers to the individual questions.
