# Supplementary material for: Pros and Cons of Immediate Sequential Bilateral Cataract Surgery from a Patient Perspective: A Survey
Source: Int J Environ Res Public Health. 2023 Jan 16;20(2):1611. doi: 10.3390/ijerph20021611 (PMC9861423; doi:10.3390/ijerph20021611)
Supplement: Supplementary file 1 [file ijerph-20-01611-s001.zip › Supplementary Tables.pdf]

Table S1. Association between the gender of the respondents and their answers to the questions from the survey. Data in the table refer to positive answers to the individual questions.

Question	Woman	Man	p
	n (%)		
**A. Questions about difficulties related to immediate sequential bilateral cataract surgery**			
Was both eyes with dressing difficult for you?	25 (20.8)	23 (30.7)	0.168
Was poor vision in the both eyes immediately after surgery difificult for you?	24 (20.0)	24 (32.0)	0.085
Was necessity to sleep on the back after surgery difficult for you?	43 (35.8)	21 (28.0)	0.329
Was necessity of applying drops to both eye at once difficult for you?	20 (16.7)	14 (18.7)	0.870
Was inability to read shortly after surgery difificult for you?	37 (30.8)	17 (22.7)	0.282
Was inability to drive shortly after surgery difficult for you?	5 (4.1)	3 (4.0)	0.720
Was both eye lacrimation or irritation difficult for you?	4 (3.3)	2 (2.6)	0.890
There were any difficulties	18 (15.0)	12 (16.0)	>0.999
**B. Questions about benefis of immediate sequential bilateral cataract surgery**			
Whether one stay in the operating room (one stress) was a benefit for you?	93 (77.5)	68 (90.7)	**0.030**
Whether limiting the number of control visits in hospital was a benefit for you?	70 (58.3)	52 (69.3)	0.164
Whether fewer visits in hospital during COVID-19 pandemic a benefit for you?	48 (40.0)	31 (41.3)	0.972
Whether quick recovery good vision in both eye was a benefit for you?	77 (64.2)	42 (56.0)	0.324
Whether quick selection of glasses for reading was a benfit for you?	43 (35.8)	20 (26.7)	0.240
Whether prospect of quick return to professional activity (work) was a benfit for you?	11 (9.2)	10 (13.3)	0.499
Whether quick return to daily activities, computer use, driving was a benefit for you?	21 (17.5)	24 (32.0)	**0.031**

Whether avoiding anisemetropia after surgery was a benefit for you?	28 (23.3)	7 (9.3)	**0.022**
Whether cost effectiveness was a benefit for you?	25 (20.8)	21 (28.0)	0.330
Whether saving time of family/friends was a benefit for you?	63 (52.5)	44 (58.7)	0.488

Table S2. Association between age of the respondents and their answers to the questions from the survey.

Question	Age<br>Median +/- IQR	p
**A. Questions about difficulties related to immediate sequential bilateral cataract surgery**		
Was both eyes with dressing difficult for you?<br>No<br>Yes	74.00 ± 13.00<br>74.00 ± 13.25	0.942
Was poor vision in the both eyes immediately after surgery difificult for you?<br>No<br>Yes	72.00 ± 14.50<br>77.00 ± 12.00	**0.002**
Was necessity to sleep on the back after surgery difficult for you?<br>No<br>Yes	73.00 ± 14.00<br>74.5 ± 12.00	0.647
Was necessity of applying drops to both eye at once difficult for you?<br>No<br>Yes	73.00 ± 15.00<br>76.00 ± 8.75	**0.043**
Was inability to read shortly after surgery difificult for you?<br>No<br>Yes	74.00 ± 13.00<br>74.00 ± 17.5	0.094
Was inability to drive shortly after surgery difficult for you?<br>No<br>Yes	73.00 ± 14.00<br>74.5 ± 13.00	0.613
Was both eye lacrimation or irritation difficult for you?<br>No<br>Yes	74.00 ± 13.00<br>74.00 ± 13.00	>0.999
There were any difficulties<br>No<br>Yes	74.00 ± 14.00<br>72.00 ± 11.75	0.456
**B. Questions about benefis of immediate sequential bilateral cataract surgery**		
Whether one stay in the operating room (one stress) was a benefit for you?<br>No	67.00 ± 17.00<br>75.00 ± 12.00	**0.001**

Yes		
Whether limiting the number of control visits in hospital was a benefit for you?<br>No<br>Yes	69.00 ± 15.00<br>75.50 ± 12.75	**<0.001**
Whether fewer visits in hospital during COVID-19 pandemic a benefit for you?<br>No<br>Yes	72.00 ± 14.00<br>76.00 ± 12.5	**0.007**
Whether quick recovery good vision in both eye was a benefit for you?<br>No<br>Yes	76.50 ± 12.00<br>72.00 ± 13.00	**0.001**
Whether quick selection of glasses for reading was a benfit for you?<br>No<br>Yes	75.00 ± 12.00<br>70.00 ± 17.50	**0.001**
Whether prospect of quick return to professional activity (work) was a benfit for you?<br>No<br>Yes	75.00 ± 12.00<br>59.00 ± 10.00	**<0.001**
Whether quick return to daily activities, computer use, driving was a benefit for you?<br>No<br>Yes	75.00 ± 12.75<br>69.00 ± 15.00	**0.002**
Whether avoiding anisemetropia after surgery was a benefit for you?<br>No<br>Yes	75.00 ± 12.00<br>66.00 ± 16.00	**<0.001**
Whether cost effectiveness was a benefit for you?<br>No<br>Yes	74.00 ± 14.00<br>76.00 ± 12.75	0.224
Whether saving time of family/friends was a benefit for you?<br>No<br>Yes	72.00 ± 14.25<br>75.00 ± 12.50	**0.001**

IQR – interquartile range

Table S3. Association between themarital status of the respondents and their answers to the questions from the survey. Data in the table refer to positive answers to the individual questions.

Question	Unmarried	Married	Widowed	p
	n (%)			
**A. Questions about difficulties related to immediate sequential bilateral cataract surgery**				
Was both eyes with dressing difficult for you?	1 (20.0)	25 (21.4)	22 (30.1)	0.338

Was poor vision in the both eyes immediately after surgery dificult for you?	0 (0.0)	29 (24.8)	19 (26.0)	0.553
Was necessity to sleep on the back after surgery difficult for you?	1 (20.0)	37 (31.6)	26 (35.6)	0.756
Was necessity of applying drops to both eye at once difficult for you?	2 (40.0)	18 (15.4)	14 (19.2)	0.245
Was inability to read shortly after surgery dificult for you?	1 (20.0)	35 (29.9)	18 (24.7)	0.773
Was inability to drive shortly after surgery difficult for you?	0 (0.0)	5 (4.3)	3 (4.1)	0.990
Was both eye lacrimation or irritation difficult for you?	0 (0.0)	3 (2.6)	3 (4.1)	0.147
There were any difficulties	0 (0.0)	20 (17.1)	10 (13.7)	0.677
**B. Questions about benefis of immediate sequential bilateral cataract surgery**				
Whether one stay in the operating room (one stress) was a benefit for you?	5 (100.0)	90 (76.9)	66 (90.4)	**0.037**
Whether limiting the number of control visits in hospital was a benefit for you?	5 (100.0)	67 (57.3)	50 (68.5)	0.060
Whether fewer visits in hospital during COVID-19 pandemic a benefit for you?	5 (100.0)	39 (33.3)	35 (47.9)	**0.002**
Whether quick recovery good vision in both eye was a benefit for you?	5 (100.0)	72 (61.5)	42 (57.5)	0.207
Whether quick selection of glasses for reading was a benfit for you?	2 (40.0)	37 (31.6)	24 (32.9)	0.874
Whether prospect of quick return to professional activity (work) was a benfit for you?	1 (20.0)	17 (14.5)	3 (4.1)	**0.034**
Whether quick return to daily activities, computer use, driving was a benefit for you?	2 (40.0)	35 (29.9)	8 (11.0)	**0.003**
Whether avoiding anisemetropia after surgery was a benefit for you?	1 (20.0)	26 (22.2)	8 (11.0)	0.103
Whether cost effectiveness was a benefit for you?	2 (40.0)	25 (21.4)	17 (23.3)	**0.018**
Whether saving time of family/friends was a benefit for you?	5 (100.0)	57 (48.7)	45 (61.6)	**0.024**

Table S4. Association between the household of the respondents and their answers to the questions from the survey. Data in the table refer to positive answers to the individual questions.

Question	Living with family	Living alone	Living with partner	p
	n (%)			
**A. Questions about difficulties related to immediate sequential bilateral cataract surgery**				
Was both eyes with dressing difficult for you?	33 (22.6)	15 (34.1)	0 (0.0)	0.155
Was poor vision in the both eyes immediately after surgery dificicult for you?	37 (25.3)	9 (20.5)	2 (40.0)	0.503
Was necessity to sleep on the back after surgery difficult for you?	53 (36.3)	10 (22.7)	1 (20.0)	0.189
Was necessity of applying drops to both eye at once difficult for you?	25 (17.1)	7 (15.9)	2 (40.0)	0.349
Was inability to read shortly after surgery dificicult for you?	37 (25.3)	15 (34.1)	2 (40.0)	0.361
Was inability to drive shortly after surgery difficult for you?	7 (4.1)	1 (4.5)	0 (0.0)	0.890
Was both eye lacrimation or irritation difficult for you?	5 (3.4)	1 (2.3)	0 (0.0)	0.107
There were any difficulties	23 (15.8)	7 (15.9)	0 (0.0)	>0.999
**B. Questions about benefis of immediate sequential bilateral cataract surgery**				
Whether one stay in the operating room (one stress) was a benefit for you?	66 (90.4)	38 (86.4)	5 (100.0)	0.550
Whether limiting the number of control visits in hospital was a benefit for you?	50 (68.5)	25 (56.8)	4 (80.0)	0.573
Whether fewer visits in hospital during COVID-19 pandemic a benefit for you?	35 (47.9)	21 (47.7)	3 (60.0)	0.325
Whether quick recovery good vision in both eye was a benefit for you?	42 (57.5)	28 (63.6)	3 (60.0)	0.905
Whether quick selection of glasses for reading was a benfit for you?	24 (32.9)	17 (38.6)	2 (40.0)	0.541

Whether prospect of quick return to professional activity (work) was a benfit for you?	3 (4.1)	2 (4.5)	1 (20.0)	0.207
Whether quick return to daily activities, computer use, driving was a benefit for you?	8 (11.0)	8 (18.2)	1 (20.0)	0.764
Whether avoiding anisemetropia after surgery was a benefit for you?	8 (11.0)	6 (13.6)	1 (20.0)	0.614
Whether cost effectiveness was a benefit for you?	17 (23.3)	15 (34.1)	3 (60.0)	**0.020**
Whether saving time of family/friends was a benefit for you?	45 (61.6)	23 (52.3)	5 (100.0)	0.130

Table S5. Association between the place of residence of the respondents and their answers to the questions from the survey. Data in the table refer to positive answers to the individual questions.

Question	Village	Small city	Medium city	Big city	Very big city	p
	n (%)					
**A. Questions about difficulties related to immediate sequential bilateral cataract surgery**						
Was both eyes with dressing difficult for you?	18 (25.7)	8 (19.5)	2 (15.4)	20 (29.0)	0 (0.0)	0.730
Was poor vision in the both eyes immediately after surgery difificult for you?	20 (28.6)	11 (26.8)	4 (30.8)	12 (17.4)	1 (50.0)	0.344
Was necessity to sleep on the back after surgery difficult for you?	26 (37.1)	14 (34.1)	1 (7.7)	22 (31.9)	1 (50.0)	0.260
Was necessity of applying drops to both eye at once difficult for you?	18 (25.7)	5 (12.2)	0 (0.0)	10 (14.5)	1 (50.0)	0.059
Was inability to read shortly after surgery difificult for you?	14 (20.0)	12 (29.3)	4 (30.8)	24 (34.8)	0 (0.0)	0.333
Was inability to drive shortly after surgery difficult for you?	3 (4.2)	2 (4.8)	0 (0.0)	3 (4.3)	0 (0.0)	0.890
Was both eye lacrimation or irritation difficult for you?	2 (2.8)	1 (2.4)	0 (0.0)	2 (2.9)	0 (0.0)	0.789

There were any difficulties	10 (14.3)	4 (9.8)	7 (53.8)	8 (11.6)	1 (50.0)	**0.003**
**B. Questions about benefis of immediate sequential bilateral cataract surgery**						
Whether one stay in the operating room (one stress) was a benefit for you?	64 (91.4)	33 (80.5)	12 (92.3)	50 (72.5)	2 (100.0)	**0.038**
Whether limiting the number of control visits in hospital was a benefit for you?	49 (70.0)	27 (65.9)	7 (53.8)	37 (53.6)	2 (100.0)	0.222
Whether fewer visits in hospital during COVID-19 pandemic a benefit for you?	33 (47.1)	17 (41.5)	4 (30.8)	24 (34.8)	1 (50.0)	0.568
Whether quick recovery good vision in both eye was a benefit for you?	42 (60.0)	20 (48.8)	8 (6150)	48 (69.6)	1 (50.0)	0.254
Whether quick selection of glasses for reading was a benfit for you?	19 (27.1)	9 (22.0)	7 (53.8)	28 (40.6)	0 (0.0)	0.070
Whether prospect of quick return to professional activity (work) was a benfit for you?	1 (1.4)	3 (7.3)	2 (15.4)	15 (21.7)	0 (0.0)	**0.001**
Whether quick return to daily activities, computer use, driving was a benefit for you?	13 (18.6)	5 (12.2)	2 (15.4)	24 (34.8)	1 (50.0)	**0.030**
Whether avoiding anisemetropia after surgery was a benefit for you?	6 (8.6)	7 (17.1)	1 (7.7)	21 (30.4)	0 (0.0)	**0.012**
Whether cost effectiveness was a benefit for you?	23 (32.9)	12 (29.3)	4 (30.8)	7 (10.1)	2 (100.0)	**0.009**
Whether saving time of family/friends was a benefit for you?	46 (65.7)	27 (65.9)	8 (61.5)	24 (34.8)	0 (0.0)	**0.001**

Table S6. Association between the education of the respondents and their answers to the questions from the survey. Data in the table refer to positive answers to the individual questions.

Question	Primary	Vocational	Secondary	Higher	p
	n (%)				

**A. Questions about difficulties related to immediate sequential bilateral cataract surgery**					
Was both eyes with dressing difficult for you?	12 (32.4)	12 (24.5)	14 (21.2)	10 (23.3)	0.643
Was poor vision in the both eyes immediately after surgery difificult for you?	15 (40.5)	12 (24.5)	14 (21.2)	7 (16.3)	0.069
Was necessity to sleep on the back after surgery difficult for you?	9 (24.3)	19 (38.8)	19 (28.8)	17 (39.5)	0.339
Was necessity of applying drops to both eye at once difficult for you?	13 (35.1)	6 (12.2)	12 (18.2)	3 (7.0)	**0.007**
Was inability to read shortly after surgery difificult for you?	9 (24.3)	9 (18.4)	20 (30.3)	16 (37.2)	0.212
Was inability to drive shortly after surgery difficult for you?	1 (2.7)	2 (4.0)	3 (4.5)	2 (4.6)	0.156
Was both eye lacrimation or irritation difficult for you?	1 (2.7)	2 (4.0)	2 (3.0)	1 (2.3)	0.587
There were any difficulties	5 (13.5)	6 (12.2)	12 (18.2)	7 (16.3)	0.827
**B. Questions about benefis of immediate sequential bilateral cataract surgery**					
Whether one stay in the operating room (one stress) was a benefit for you?	32 (86.5)	44 (89.8)	54 (81.8)	31 (72.1)	0.140
Whether limiting the number of control visits in hospital was a benefit for you?	27 (73.0)	32 (65.3)	41 (62.1)	22 (51.2)	0.325
Whether fewer visits in hospital during COVID-19 pandemic a benefit for you?	18 (48.6)	20 (40.8)	26 (39.4)	15 (34.9)	0.655
Whether quick recovery good vision in both eye was a benefit for you?	22 (59.5)	24 (49.0)	42 (63.6)	31 (72.1)	0.143
Whether quick selection of glasses for reading was a benfit for you?	11 (29.7)	7 (14.3)	26 (39.4)	19 (44.2)	**0.009**
Whether prospect of quick return to professional activity (work) was a benfit for you?	0 (0.0)	0 (0.0)	15 (22.7)	6 (14.0)	**<0.001**
Whether quick return to daily activities, computer use, driving was a benefit for you?	0 (0.0)	9 (18.4)	22 (33.3)	14 (32.6)	**<0.001**

Whether avoiding anisemetropia after surgery was a benefit for you?	2 (5.4)	7 (14.3)	14 (21.2)	12 (27.9)	0.051
Whether cost effectiveness was a benefit for you?	18 (48.6)	11 (22.4)	14 (21.2)	3 (7.0)	**<0.001**
Whether saving time of family/friends was a benefit for you?	26 (70.3)	31 (63.3)	32 (48.5)	18 (41.9)	**0.030**

Table S7. Association between theindependence in self-care of the respondents and their answers to the questions from the survey. Data in the table refer to positive answers to the individual questions.

Question	Fully independent	Partly care-dependent	Fully care-dependent	p
	n (%)			
**A. Questions about difficulties related to immediate sequential bilateral cataract surgery**				
Was both eyes with dressing difficult for you?	36 (27.9)	7 (16.3)	5 (21.7)	0.291
Was poor vision in the both eyes immediately after surgery dificult for you?	24 (18.6)	12 (27.9)	12 (52.2)	**0.002**
Was necessity to sleep on the back after surgery difficult for you?	43 (33.3)	14 (32.6)	7 (30.4)	0.963
Was necessity of applying drops to both eye at once difficult for you?	20 (15.5)	11 (25.6)	3 (13.0)	0.299
Was inability to read shortly after surgery dificult for you?	36 (27.9)	14 (32.6)	4 (17.4)	0.421
Was inability to drive shortly after surgery difficult for you?	5 (6.9)	3 (7.0)	0 (0.0)	0.708
Was both eye lacrimation or irritation difficult for you?	4 (3.1)	1 (2.3)	1 (4.3)	0.077
There were any difficulties	19 (14.7)	5 (11.6)	6 (23.1)	0.321
**B. Questions about benefis of immediate sequential bilateral cataract surgery**				
Whether one stay in the operating room (one stress) was a benefit for you?	102 (79.1)	38 (88.4)	21 (91.3)	0.252

Whether limiting the number of control visits in hospital was a benefit for you?	73 (56.6)	30 (69.8)	19 (82.6)	**0.032**
Whether fewer visits in hospital during COVID-19 pandemic a benefit for you?	50 (38.3)	16 (37.2)	13 (56.5)	0.246
Whether quick recovery good vision in both eye was a benefit for you?	85 (65.9)	22 (51.2)	12 (52.2)	0.150
Whether quick selection of glasses for reading was a benfit for you?	45 (34.9)	15 (34.9)	3 (13.0)	0.109
Whether prospect of quick return to professional activity (work) was a benfit for you?	20 (15.5)	0 (0.0)	1 (4.3)	**0.004**
Whether quick return to daily activities, computer use, driving was a benefit for you?	38 (29.5)	5 (11.6)	2 (8.7)	**0.012**
Whether avoiding anisemetropia after surgery was a benefit for you?	29 (22.5)	4 (9.3)	2 (8.7)	0.077
Whether cost effectiveness was a benefit for you?	27 (20.9)	11 (25.6)	8 (34.8)	0.333
Whether saving time of family/friends was a benefit for you?	57 (44.2)	34 (79.1)	16 (69.6)	**<0.001**

Table S8. Association between the respondents who drive/don`t drive a car and their answers to questions from the survey. Data in the table refer to positive answers to the individual questions.

Question	Driving a car	Not driving a car	Occasionally driving a car	p
	n (%)			
**A. Questions about difficulties related to immediate sequential bilateral cataract surgery**				
Was both eyes with dressing difficult for you?	13 (23.6)	32 (24.2)	3 (42.9)	0.536
Was poor vision in the both eyes immediately after surgery difificult for you?	11 (20.0)	37 (28.0)	0 (0.0)	0.183
Was necessity to sleep on the back after surgery difficult for you?	16 (29.1)	46 (34.8)	1 (14.3)	0.473
Was necessity of applying drops to both eye at once difficult for you?	7 (12.7)	24 (18.2)	2 (28.6)	0.379

Was inability to read shortly after surgery difificult for you?	19 (34.5)	34 (25.8)	1 (14.3)	0.397
Was both eye lacrimation or irritation difficult for you?	2 (3.6)	4 (3.0)	0 (0.0)	0.577
There were any difficulties	9 (16.4)	19 (14.4)	2 (28.6)	0.446
**B. Questions about benefis of immediate sequential bilateral cataract surgery**				
Whether one stay in the operating room (one stress) was a benefit for you?	40 (72.7)	115 (87.1)	5 (71.4)	**0.038**
Whether limiting the number of control visits in hospital was a benefit for you?	24 (43.6)	91 (68.9)	6 (85.7)	**0.002**
Whether fewer visits in hospital during COVID-19 pandemic a benefit for you?	16 (29.1)	58 (43.9)	4 (57.1)	0.107
Whether quick recovery good vision in both eye was a benefit for you?	37 (67.3)	76 (57.6)	5 (71.4)	0.415
Whether quick selection of glasses for reading was a benfit for you?	21 (38.2)	37 (28.0)	4 (57.1)	0.126
Whether prospect of quick return to professional activity (work) was a benfit for you?	16 (29.1)	4 (3.0)	1 (14.3)	**<0.001**
Whether quick return to daily activities, computer use, driving was a benefit for you?	30 (54.5)	13 (9.8)	2 (28.6)	**<0.001**
Whether avoiding anisemetropia after surgery was a benefit for you?	13 (23.6)	22 (16.7)	0 (0.0)	0.295
Whether cost effectiveness was a benefit for you?	7 (12.7)	36 (27.3)	3 (42.9)	**0.033**
Whether saving time of family/friends was a benefit for you?	18 (32.7)	84 (63.6)	4 (57.1)	**<0.001**

Table S9. Association between the respondents who use/ not use a computer and their answers to the questions from the survey. Data in the table refer to positive answers to the individual questions.

Question	Not using a computer	Using a computer	p
	n (%)		

**A. Questions about difficulties related to immediate sequential bilateral cataract surgery**			
Was both eyes with dressing difficult for you?	31 (24.8)	17 (24.3)	>0.999
Was poor vision in the both eyes immediately after surgery difificult for you?	38 (30.4)	10 (14.3)	**0.020**
Was necessity to sleep on the back after surgery difficult for you?	40 (32.0)	24 (34.3)	0.867
Was necessity of applying drops to both eye at once difficult for you?	28 (22.4)	6 (8.6)	**0.025**
Was inability to read shortly after surgery difificult for you?	31 (24.8)	23 (32.9)	0.299
Was inability to drive shortly after surgery difficult for you?	5 (4.0)	3 (4.2)	0.872
Was both eye lacrimation or irritation difficult for you?	4 (3.2)	2 (2.8)	0.690
There were any difficulties	19 (15.2)	11 (15.7)	>0.999
**B. Questions about benefis of immediate sequential bilateral cataract surgery**			
Whether one stay in the operating room (one stress) was a benefit for you?	108 (86.4)	53 (75.7)	0.091
Whether limiting the number of control visits in hospital was a benefit for you?	84 (67.2)	38 (54.3)	0.102
Whether fewer visits in hospital during COVID-19 pandemic a benefit for you?	54 (43.2)	25 (35.7)	0.385
Whether quick recovery good vision in both eye was a benefit for you?	70 (56.0)	49 (70.0)	0.077
Whether quick selection of glasses for reading was a benfit for you?	28 (22.4)	35 (50.0)	**<0.001**
Whether prospect of quick return to professional activity (work) was a benfit for you?	3 (2.4)	18 (25.7)	**<0.001**
Whether quick return to daily activities, computer use, driving was a benefit for you?	14 (11.2)	31 (44.3)	**<0.001**
Whether avoiding anisemetropia after surgery was a benefit for you?	13 (10.4)	22 (31.4)	**0.001**
Whether cost effectiveness was a benefit for you?	37 (29.6)	9 (12.9)	**0.014**
Whether saving time of family/friends was a benefit for you?	76 (60.8)	31 (44.3)	**0.038**

Table S10. Association between the respondents who use/ not use a mobile phone and their answers to the questions from the survey. Data in the table refer to positive answers to the individual questions.

Question	Not using a mobile phone	Using a mobile phone	p
	n (%)		
**A. Questions about difficulties related to immediate sequential bilateral cataract surgery**			
Was both eyes with dressing difficult for you?	17 (23.6)	31 (25.2)	0.939
Was poor vision in the both eyes immediately after surgery difificult for you?	23 (31.9)	25 (20.3)	0.100
Was necessity to sleep on the back after surgery difficult for you?	27 (37.5)	37 (30.1)	0.365
Was necessity of applying drops to both eye at once difficult for you?	18 (25.0)	16 (13.0)	0.053
Was inability to read shortly after surgery difificult for you?	16 (22.2)	38 (30.9)	0.254
Was inability to drive shortly after surgery difficult for you?	2 (2.8)	6 (4.8)	0.192
Was both eye lacrimation or irritation difficult for you?	2 (2.8)	4 (3.2)	0.691
There were any difficulties	5 (6.9)	25 (20.3)	**0.022**
**B. Questions about benefis of immediate sequential bilateral cataract surgery**			
Whether one stay in the operating room (one stress) was a benefit for you?	61 (84.7)	100 (81.3)	0.680
Whether limiting the number of control visits in hospital was a benefit for you?	53 (73.6)	69 (56.1)	**0.022**
Whether fewer visits in hospital during COVID-19 pandemic a benefit for you?	32 (43.1)	48 (39.0)	0.688
Whether quick recovery good vision in both eye was a benefit for you?	36 (50.0)	83 (67.5)	**0.024**
Whether quick selection of glasses for reading was a benfit for you?	15 (20.8)	48 (39.0)	**0.014**

Whether prospect of quick return to professional activity (work) was a benfit for you?	2 (2.8)	19 (15.7)	**0.012**
Whether quick return to daily activities, computer use, driving was a benefit for you?	6 (8.3)	39 (31.7)	**<0.001**
Whether avoiding anisemetropia after surgery was a benefit for you?	8 (11.1)	27 (22.0)	0.087
Whether cost effectiveness was a benefit for you?	23 (31.9)	23 (18.7)	0.054
Whether saving time of family/friends was a benefit for you?	51 (70.8)	56 (45.5)	**0.001**

Table S11. Association between the respondents who read/ not read much and their answers to the questions from the survey. Data in the table refer to positive answers to the individual questions.

Question	Not read much	Read much	p
	n (%)		
**A. Questions about difficulties related to immediate sequential bilateral cataract surgery**			
Was both eyes with dressing difficult for you?	26 (22.4)	22 (27.8)	0.487
Was poor vision in the both eyes immediately after surgery difificult for you?	33 (28.4)	15 (19.0)	0.181
Was necessity to sleep on the back after surgery difficult for you?	35 (30.2)	29 (36.7)	0.424
Was necessity of applying drops to both eye at once difficult for you?	20 (17.2)	14 (17.7)	>0.999
Was inability to read shortly after surgery difificult for you?	32 (27.6)	22 (27.8)	>0.999
Was inability to drive shortly after surgery difficult for you?	5 (4.3)	3 (3.8)	0.405
Was both eye lacrimation or irritation difficult for you?	3 (2.6)	3 (3.8)	0.690
There were any difficulties	20 (17.2)	10 (12.7)	0.504
**B. Questions about benefis of immediate sequential bilateral cataract surgery**			
Whether one stay in the operating room (one stress) was a benefit for you?	95 (81.9)	66 (83.5)	0.916

Whether limiting the number of control visits in hospital was a benefit for you?	72 (62.1)	50 (63.3)	0.982
Whether fewer visits in hospital during COVID-19 pandemic a benefit for you?	41 (35.3)	38 (48.1)	0.103
Whether quick recovery good vision in both eye was a benefit for you?	64 (55.2)	55 (69.6)	0.060
Whether quick selection of glasses for reading was a benfit for you?	33 (28.4)	30 (38.0)	0.215
Whether prospect of quick return to professional activity (work) was a benfit for you?	14 (12.1)	7 (8.9)	0.635
Whether quick return to daily activities, computer use, driving was a benefit for you?	23 (19.8)	22 (27.8)	0.258
Whether avoiding anisemetropia after surgery was a benefit for you?	17 (14.7)	18 (22.8)	0.207
Whether cost effectiveness was a benefit for you?	32 (27.6)	14 (17.7)	0.155
Whether saving time of family/friends was a benefit for you?	63 (54.3)	44 (55.7)	0.965

Table S12. Association between the occupational status of the respondents and their answers to the questions from the survey. Data in the table refer to positive answers to the individual questions.

Question	Non-working pensioner	Working Pensioner	Working professionally	p
	n (%)			
**A. Questions about difficulties related to immediate sequential bilateral cataract surgery**				
Was both eyes with dressing difficult for you?	42 (26.9)	4 (25.0)	2 (8,.7	0.152
Was poor vision in the both eyes immediately after surgery difificult for you?	44 (28.2)	3 (18.8)	1 (4.3)	**0.029**
Was necessity to sleep on the back after surgery difficult for you?	53 (34.0)	4 (25.0)	7 (30.4)	0.742
Was necessity of applying drops to both eye at once difficult for you?	31 (19.9)	28 (12.5)	1 (4.3)	0.173

Was inability to read shortly after surgery difificult for you?	36 (23.1)	4 (25.0)	14 (60.9)	**0.002**
Was inability to drive shortly after surgery difficult for you?	4 (2.6)	1 (6.25)	3 (12.8)	0.061
Was both eye lacrimation or irritation difficult for you?	5 (3.2)	1 (6.25)	0 (0.0)	0.347
There were any difficulties	24 (15.4)	5 (31.2)	1 (4.3)	0.068
**B. Questions about benefis of immediate sequential bilateral cataract surgery**				
Whether one stay in the operating room (one stress) was a benefit for you?	136 (87.2)	14 (87.5)	11 (47.8)	**<0.001**
Whether limiting the number of control visits in hospital was a benefit for you?	107 (68.6)	9 (56.2)	6 (26.1)	**<0.001**
Whether fewer visits in hospital during COVID-19 pandemic a benefit for you?	69 (44.2)	7 (43.8)	3 (13.0)	**0.017**
Whether quick recovery good vision in both eye was a benefit for you?	90 (57.7)	12 (75.0)	17 (73.9)	0.161
Whether quick selection of glasses for reading was a benfit for you?	42 (26.9)	5 (31.2)	26 (69.6)	**<0.001**
Whether prospect of quick return to professional activity (work) was a benfit for you?	1 (0.6)	4 (25.0)	16 (69.6)	**<0.001**
Whether quick return to daily activities, computer use, driving was a benefit for you?	25 (16.0)	8 (50.0)	12 (52.2)	**<0.001**
Whether avoiding anisemetropia after surgery was a benefit for you?	20 (12.8)	3 (18.8)	12 (52.2)	**<0.001**
Whether cost effectiveness was a benefit for you?	44 (28.2)	0 (0.0)	2 (8.7)	**0.005**
Whether saving time of family/friends was a benefit for you?	94 (60.3)	9 (56.2)	4 (17.4)	**0.001**
